# Cooperative Integration and Representation Underlying Bilateral Network of Fly Motion-Sensitive Neurons

**DOI:** 10.1371/journal.pone.0085790

**Published:** 2014-01-23

**Authors:** Yoshinori Suzuki, Takako Morimoto, Hiroyoshi Miyakawa, Toru Aonishi

**Affiliations:** 1 Interdisciplinary Graduate School of Science and Engineering, Tokyo Institute of Technology, Yokohama, Kanagawa, Japan; 2 School of Life Sciences, Tokyo University of Pharmacy and Life Sciences, Hachio-ji, Tokyo, Japan; Tokai University, Japan

## Abstract

How is binocular motion information integrated in the bilateral network of wide-field motion-sensitive neurons, called lobula plate tangential cells (LPTCs), in the visual system of flies? It is possible to construct an accurate model of this network because a complete picture of synaptic interactions has been experimentally identified. We investigated the cooperative behavior of the network of horizontal LPTCs underlying the integration of binocular motion information and the information representation in the bilateral LPTC network through numerical simulations on the network model. First, we qualitatively reproduced rotational motion-sensitive response of the H2 cell previously reported in vivo experiments and ascertained that it could be accounted for by the cooperative behavior of the bilateral network mainly via interhemispheric electrical coupling. We demonstrated that the response properties of single H1 and Hu cells, unlike H2 cells, are not influenced by motion stimuli in the contralateral visual hemi-field, but that the correlations between these cell activities are enhanced by the rotational motion stimulus. We next examined the whole population activity by performing principal component analysis (PCA) on the population activities of simulated LPTCs. We showed that the two orthogonal patterns of correlated population activities given by the first two principal components represent the rotational and translational motions, respectively, and similar to the H2 cell, rotational motion produces a stronger response in the network than does translational motion. Furthermore, we found that these population-coding properties are strongly influenced by the interhemispheric electrical coupling. Finally, to test the generality of our conclusions, we used a more simplified model and verified that the numerical results are not specific to the network model we constructed.

## Introduction

For many living beings, binocular visual perception is one of the most important functions of their visual systems. For example, the retinal images in the eyes are slightly different from each other, which is referred to as binocular disparity, and this difference provides information that the brain can use to calculate the depth of objects in the visual field. In monkey's visual cortex has been reported to have binocular depth neurons that are tuned to different ranges of binocular disparity [1]. Besides depth perception, visual ego-motion perception is an out standing function of binocular vision. For almost every animal, including vertebrates and invertebrates, ego-motion perception plays a dominant role in their motion control [2]–[6]. The direction of optic flow fields in the eyes depends on the type of ego-motion. Yaw-rotational motion of animals (rotational ego-motion) elicits two distinct optic flows directed from front-to-back and from back-to-front on each eye. In contrast, forward or backward translation of animals (translational ego-motion) elicits an optic flow directed either from front-to-back or from back-to-front on both eyes. Motion stimuli caused by rotational ego-motion are referred to as in-phase motion stimuli, whereas ones caused by translational ego-motion are referred to as out-of-phase motion stimuli. Thus, the combination of optic flow fields in the eyes provides information that the brain can use to distinguish whether they are rotating or translating. To achieve this computation, motion information from the eyes has to be integrated in the brain. In this paper, we study the visual system of flies, an ideal model system to analyze such a binocular computation [7]–[15].

**Figure 1:**
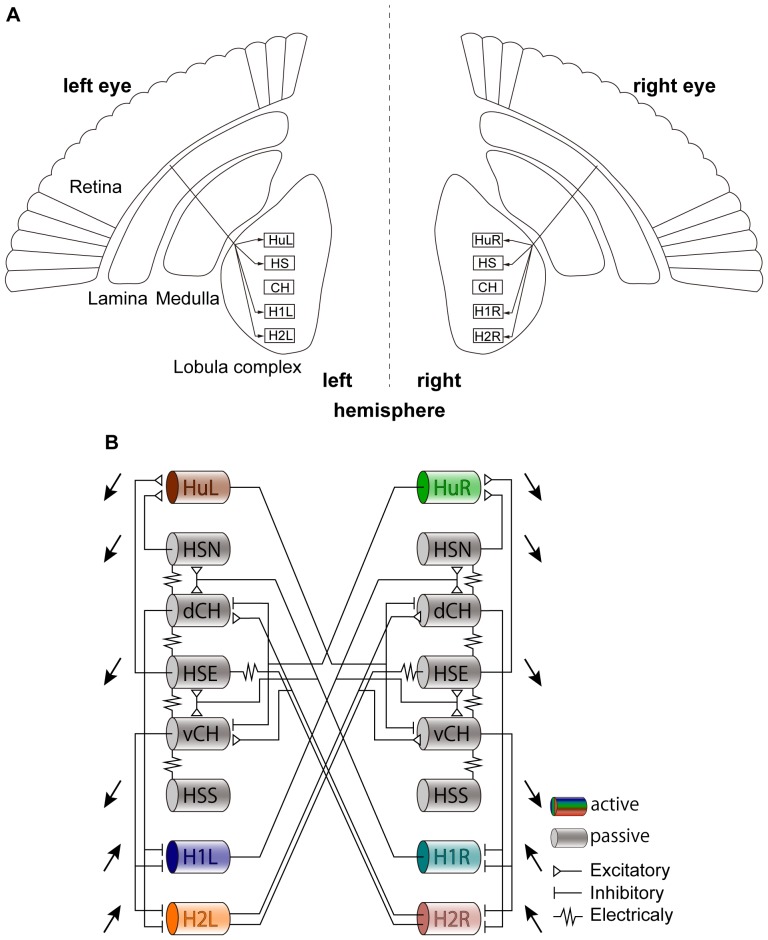
Schematic diagrams of the fly optic lobe and circuit of LPTCs with horizontal preferred directions. A: Fly visual system consisting of neuropils called the lamina, medulla, and lobula complex in the two hemispheres. Visual motion information on each side of the visual field is retinotopically processed through the lamina and medulla and converges on the lobula complex in the ipsilateral hemisphere. The complex contains a set of wide-field motion-sensitive neurons, called lobula plate tangential cells(LPTCs) [19]. B: Bilateral network of the LPTCs with horizontal preferred directions. Each hemisphere consists of eight cells: those named Hu, H1 and H2 are spiking neurons (colored), whereas the others named HS and CH are graded-potential neurons (gray). These LPTCs are mutually coupled through intrahemispheric and interhemispheric connections. Open triangles, bars and resister symbols indicate excitatory, inhibitory and electrical synapses, respectively. The cells with black arrows receive projections from the first-order neuropils, and the direction of each arrow denotes the preferred direction of each cell. dCH and vCH (without black arrows) do not directly receive projections from the first-order neuropils [11].

Motion-sensitive neurons that analyze optic flow fields and often have complex receptive fields are found at higher orders of processing in the visual systems of many species [16]–[20]. These neurons are involved in the visual perception of orientation, locomotion tasks, and head movements. The neural mechanisms underlying optic flow analysis have been studied especially well in flies. In the visual system of the blowfly *Calliphoravicina*, there are the hierarchical structures consisting of four neuropils in the left and right hemispheres, and these neuropils retinotopically process motion information from the left and right eyes, respectively. After retinotopic processing, visual information converges in the lobula complex, which subsequently receive the signal processed by the medulla (Figure 1A). It contains a set of wide-field motion-sensitive neurons called lobula plate tangential cells (LPTCs) [20], [21]. LPTCs have complex receptive fields that cover a large part of the ipsilateral visual hemi-field and show directional-selective motion responses by shifting their membrane potential as well as evoking an action potential [12], [15], [20], [22]–[25]. Some of these cells have been also found in *Drosophila* [26], [27]. The LPTCs are grouped into horizontal and vertical cells that predominantly respond to horizontal (front-to-back or back-to-front) and vertical (upward or downward) motion stimulus on the ipsilateral visual hemi-field. The LPTCs include eight horizontal cells in each hemisphere of the blowfly's brain, of which three are named HS cells (HSN, HSE and HSS [28]), two are named CH cells (dCH and vCH [29], [30]), and the others are named Hu, H1 and H2 [8], [10], [13]. The H1, H2 and Hu cells are spiking neurons, whereas HS and CH cells are graded-potential neurons.

Previous studies have identified the bilateral network of LPTCs [10], [13], [31]–[33]. It has been reported that the LPTCs make the intrahemispheric and interhemispheric connections and have the possibility to integrate the binocular motion information in the network. Some of the horizontal LPTCs have been reported to have larger responses to in-phase motion than to out-of-phase motion [8]–[10], [13], [29]. Farrow et al. (2006) [13] studied the cooperative behavior of H2 and contralateral HSE cells and demonstrated that an interhemispheric electrical coupling between the H2 cell and its contralateral HSE cell is an important factor in determining the sensitivity of the H2 cell to binocular motion stimuli. This was a pioneering study revealing that the response properties originated from not a single-cell behavior but a cooperative behavior with another LPTC in the network. However, whereas these studies focused on parts of the network, there has been no work as yet on cooperative integration of binocular motion performed by the whole LPTC network. In this paper, we reveal how the whole LPTC network works to integrate the binocular motion information and how the information are encoded by neural activity at all levels from a single cell up to the population of cells.

To address this problem, we took a mathematical modeling approach. It is technically difficult to record the membrane potentials of many cells simultaneously in vivo, and hence, it is difficult to ascertain data about the cooperative behavior of the whole network through measurements. However, it is possible to construct an accurate model of the bilateral LPTC network because a complete picture of its synaptic interactions has been experimentally identified. In this study, we focused on the network of horizontal LPTCs that mainly contributes to binocular motion integration and constructed a bilateral network model that takes into account all synaptic connections that have experimentally identified in the actual network.

First, we qualitatively reproduced the in-phase motion-sensitive response of the H2 cell that had been previously reported and made sure that it could be accounted for by the cooperative behavior of the bilateral network mainly via interhemispheric electrical coupling. We also found that the response properties of single H1 and Hu cells, unlike H2 cell, are not influenced by motion stimuli in the contralateral visual hemi-field, but that correlations between these cell activities are enhanced by the in-phase motion stimulus. Next, to reveal the coding properties of a population of spiking LPTCs, we performed principal component analysis (PCA) on the firing rates of all spiking LPTCs. We showed that the two orthogonal patterns of correlated population activities given by the first two principal components represent the in-phase and out-of-phase motions, respectively, and the population activity is more sensitive to the in-phase motion stimuli. Furthermore, we found that these population-coding properties are strongly influenced by the interhemispheric electrical coupling. Finally, by reproducing these population-coding properties with a reduced model, we confirmed that the numerical results are not specific to the network model we constructed.

## Materials and Methods

### Fly visual system

The fly visual system consists of four neuropils called the lamina, medulla, lobula, and lobula plate that exhibit the same columnar structure as the retina and are retinotopically organized in both hemispheres. Visual motion information from each side of the visual field is retinotopically processed and converges on the lobula complex comprised of the lobula and lobula plate (Figure 1A). This complex contains a set of large motion-sensitive neurons, called lobula plate tangential cells (LPTCs). A total of 60 different cells exist in the blowfly, all of which show directional-selective motion responses by shifting their membrane potential as well as their action potentials [20]. During preferred direction (PD) motion stimulation, the cells in each hemisphere are depolarized or generate action potentials, whereas during antipreferred or null direction (ND) motion stimulation, the cells in each hemisphere are hyperpolarized.

### Bilateral horizontal LPTCs network

The LPTCs are grouped into horizontal and vertical cells that predominantly respond to horizontal and vertical motion stimuli, respectively. As mentioned above, we will focus on the horizontal cells. Each hemisphere consists of eight horizontal cells (Figure 1B). H1, H2 and Hu cells produce action potentials during PD motion stimulations (i.e., spiking cells). HS and CH cells respond to PD motion stimuli in a graded way (i.e., graded-potential cells). These horizontal LPTCs are mutually coupled through intrahemispheric and interhemispheric connections with various electrical and chemical synapses, as shown in Figure 1B. The experimental findings on these couplings have been reported in [10], [13], [15], [20]. We constructed a network model of horizontal LPTCs by mainly referring to Borst et al. (2011) [15].

### Conductance-based model (Detailed model)

To keep the model relatively simple, we decided that the morphology of each model cell would be a simple long cylinder. Here, the model cells do not have dendritic branches as in the real cells. The HS and CH cells are modeled as one passive compartment (gray cylinders in Figure 1B), and the H1, H2 and Hu cells are modeled as one active compartment capable of producing action potentials (colored cylinders in Figure 1B). We ascertained that the behaviors of the single compartment model are much the same as the ones of the multi compartment model (data not shown). The morphological parameters of each model cell are listed in Table 1. The properties and distribution of ion-conductances in LPTCs are still unknown (but see also [23], [34]). Thus, instead of high-dimensional conductance-based models, the type-I Morris-Lecar (ML) model is used to describe membrane currents in the active compartments. The ML model is one of the simplest conductance-based models capable of reproducing the variety of oscillatory behaviors found in various excitatory membranes [35]. The ML model and simple passive model used here are uniformly described in terms of the following ordinary differential equations:
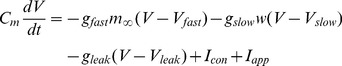
(1)

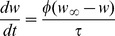
where the conductances 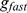, 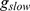 and 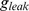 are for the fast, slow and the leak channels, respectively. Note that when 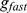 and 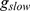 are zero, these equations are equivalent to a simple passive model. The passive electrical parameters including the leak parameters of the ML model are listed in Table 1. In this paper, the fast and slow channels are not specified as specific ion channels, whereas in the original ML model, the fast and slow channels are respectively calcium and potassium channels. The functions 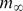 and 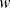 are the equilibrium open fractions for the fast and slow channels, and 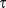 is the activation time constant for the slow channel. These functions are
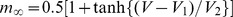


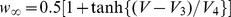


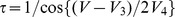
The parameters of the type-I ML model are listed in Table 2. 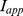 denotes the total postsynaptic current from motion-sensitive cells in the first-order neuropils (medulla and lobula), and 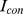 is the total postsynaptic current from the other horizontal LPTCs. A detailed explanation of 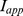 and 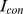 is given in the following subsections. Numerical simulations were carried out with the NEURON simulator.

**Table 1:** Morphological parameters and passive electrical parameters of each.

Cell type	Length 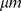	Diameter 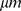	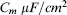	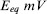	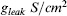
H1	1000	5	20		0.002
H2	200	5	20		0.002
Hu	200	5	20		0.002
HS	250	15	1	−50	0.001
CH	250	15	1	−50	0.001

**Table 2:** Parameters of the type-I Morris-Lecar model.

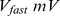	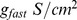	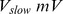	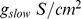	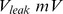
120	0.004	−84	0.008	−60
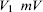	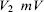	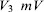	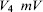	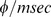
−1.2	18	12	17.4	0.066

### Mimicking visual stimuli

We used four different binocular motion stimuli: clockwise(C), counterclockwise(CC), front-to-back(FB) and back-to-front(BF). The clockwise and counterclockwise motion stimuli are classified as in-phase horizontal binocular motion stimuli that are elicited when a fly rotates about its vertical body axis (yaw). The front-to-back and back-to-front stimuli are classified as out-of-phase horizontal binocular motion stimuli that are elicited by forward and backward translation. By using these four motion stimuli, we can verify the response properties of the horizontal LPTCs network for all possible combinations of directions of stimuli for each eye. A simple way to simulate the responses of the LPTCs to the stimuli is to mimic the total postsynaptic current from the earlier neuropils, 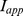, with either a depolarizing or hyperpolarizing DC current depending on whether each stimulus direction is preferred or not by each cell. In this way, 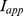 is determined by the following formula:
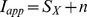


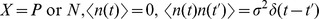
where 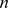 is a white noise term independent from neuron to neuron and 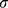 is the noise intensity. 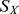 represents the DC signal that changes to 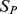 or 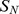 depending on whether each motion stimulus is PD or ND for each cell. The cells with black arrows in Figure 1B receive projections from the first-order neuropils, and the direction of each arrow denotes the preferred direction of each cell. Table 3 shows the amplitude of the DC signal given to each horizontal LPTC. Note that the CH cells (not marked with black arrows in Figure 1B) do not directly receive projections from the earlier neuropils [10], and thus, 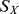 of the CH cells is set to zero. Table 4 shows all combinations of either depolarizing or hyperpolarizing current for representing the four stimuli.

**Table 3:** Amplitude of the DC signal corresponding to PD and ND motion stimulus.

	H1	H2	HS
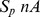	10	2	3.6
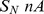	−1	−0.2	−1.3

**Table 4:** Combinations of either depolarizing or hyperpolarizing current for representing the four types of stimuli.

	C	CC	FB	BF
H1L	P	N	N	P
H2L	P	N	N	P
HuL	N	P	P	N
HSL	N	P	P	N
H1R	N	P	N	P
H2R	N	P	N	P
HuR	P	N	P	N
HSR	P	N	P	N

To measure the robustness of neural coding, we define the signal to noise ratio (SNR) for 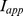 as
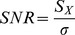
We analyzed the responses of spiking LPTCs with various SNR.

### Connection properties

To construct a network model of the horizontal LPTCs, we connect the conductance-based models through electrical and chemical synapses, as shown in Figure 1B. In Eq. (1), 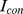 denotes the total postsynaptic current from the other horizontal LPTCs. The implementation of 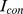 can be easily realized with the NEURON simulator. Electrical couplings can be implemented with the NMODL function of the NEURON simulator. This function makes a conductive connection between two connected compartments with a particular conductance. In this study, all electrical couplings have the same conductance (40 nS) [13]. The excitatory and inhibitory chemical synapses are modeled as a change in synaptic conductivities triggered by spike events in presynaptic cells, which are implemented using the ExpSyn function of the NEURON simulator. The reversal potentials of the excitatory and inhibitory synapses are 0 mV and -70 mV, respectively. The conductance of excitatory and inhibitory synapses is described by a simple exponential decay with a time constant of 0.3 ms and amplitudes of 0.01and 0.005 nS, respectively. We manually tuned these synaptic parameters to fit the simulated EPSP and IPSP to previously reported physiological data [10], [13].

As described above, the CH and HS cells are non-spiking. It has been reported that the CH cell inhabits the activities of the ipsilateral H1 and H2 cells, and the HS cell excites the activities of the ipsilateral Hu cell. In this study, the excitatory postsynaptic current depending on the presynaptic graded-potential is described using a sigmoid function,
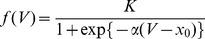
(2)where 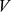 is the graded potential of the presynaptic neuron, i.e., the HS cell. The inhibitory postsynaptic current depending on the presynaptic graded potential is given by 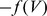. The parameters used in the simulations were 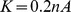, 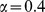 and 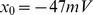.

The purpose of this study is to clarify the role of the bilateral horizontal LPTC network in the binocular motion integration. For this purpose, we compared two cases: one in which the cells are mutually connected as described above, the other in which the cells are isolated from each other without lateral connections. In the following sections, we will refer to these cases as the connected case and disconnected case.

### Formal neuron model (Reduced model)

To check the generality and robustness of the results obtained from the conductance-based model, we constructed a reduced model and verified whether or not it qualitatively reproduced the results. In this model, five graded-potential cells, which are coupled through electrical synapses and have similar response properties, are merged into a single neuron named HS/CH. Furthermore, we used McCulloch Pitts formal neurons instead of the conductance-based model. The state space equation for the reduced model is
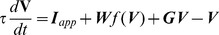
(3)

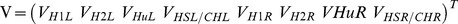


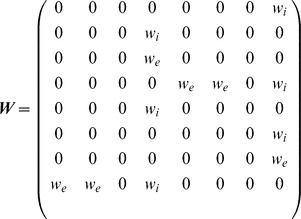


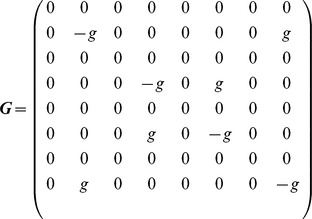
where 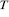 denotes matrix transposition, 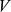 denotes the state vector consisting of the membrane potentials of eight cells and 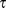 is the time constant (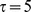 msec). 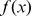 is the sigmoidal function defined in Eq. (2), which represents the transfer function of each cell. The matrix 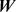 represents the couplings between these cells through chemical synapses, and the matrix 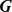 denotes the electrical couplings between them. 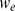 and 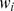 in 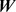 are the weights of the excitatory and inhibitory synapses, respectively, and 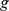 in 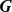 is the conductance of the electrical couplings. Thus, each component of the vector 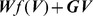 describes the total postsynaptic current in each cell via lateral connections.

The vector 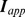 in the state space equation denotes the input vector in which each component represents the total postsynaptic current of each cell from motion-sensitive cells in the earlier neuropils. As described in the detailed model, to simply simulate responses of the LPTCs to the four types of stimuli, 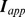 is mimicked with a combination of depolarizing or hyperpolarizing DC currents depending on whether each stimulus direction is preferred or not by each cell. 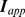 is given by
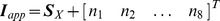


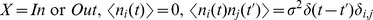
where 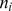 are white noise term independent from neuron to neuron and 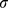 is the noise intensity. 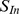 and 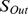 are the input vectors when presenting in-phase and out-of-phase stimuli:
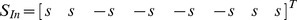


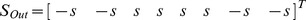
where 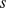 is a time-dependent variable taking +1 or -1. If 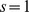, the input patterns of 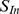 and 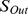 correspond to clockwise rotation and forward translation motion stimuli, respectively, whereas if 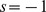, the input patterns of 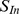 and 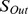 correspond to counterclockwise rotation and backward translation motion stimuli, respectively.

The parameters for the formal neuron model used in the simulations were 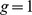, 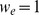, 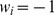, 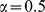, 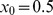 and 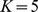. The simulations were carried out with MATLAB.

### Data analysis

#### Cross-correlation analysis

Let 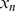 and 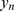 be the mean firing rates of different cells at time 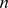. The cross-correlation between 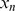 and 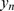 is defined as
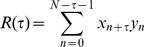
where 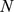 is the length of these two sequences and 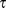 is a time lag. For the cross-correlation analyses, the mean firing rate was calculated at intervals of 10 msec.

#### Principal component analysis of multi-neuronal activity

The principal component analysis (PCA) is a dimensionality reduction technique in which high-dimensional data is linearly projected on an orthogonal subspace spanned by vectors representing highly correlated directions [36]. We tried to elucidate the correlated activities of a neural population coding the four types of binocular motion stimuli by applying PCA to simulated multi-neuronal activity data. First, we calculated the mean firing rates of six spiking LPTCs at intervals of 150 msec within non-overlapping 150 msec temporal windows, and we constructed a set of six-dimensional firing rate vectors, 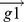, 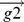, 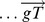, where 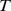 denotes the total number of firing rate vectors (e.g. 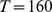 when the length of the spike sequence is 24 sec). Next, we performed principal component analysis (PCA) on the set of the firing rate vectors. The calculation was carried out with the princomp function of MATLAB.

## Results

### The activity of the H2 cell in response to its PD motion stimuli is only modified by the contralateral LPTC activities

We carried out numerical simulations of the detailed model (see Material and Methods for details) to analyze how the activity of single LPTCs of one hemisphere in response to the PD motion stimulus presented in the ipsilateral visual hemi-field are modified by changes in the contralateral LPTCs activities depending on motion stimuli in the contralateral visual hemi-field. We focused on three spiking LPTCs in the left hemisphere, H1L, H2L and HuL. The H2L cell is directly connected with the HSE cell of the right hemisphere through an interhemispheric electrical coupling, and the H1L and HuL cells indirectly receive effects from the contralateral cells via other ipsilateral cells (see Figure 1B). Note that it is not necessary to show the activities of counterparts of these three cells in the right hemisphere. This is because the bilateral LPTCs network has a reflective symmetric structure, statistical response properties of LPTCs separately located on both hemispheres to pair of binocular motion stimuli which are in symmetric relation are the same. Therefore, it is sufficient to check for the responses of LPTCs located on one hemisphere.

To quantify the effect of the connections among LPTCs on the activities of each spiking LPTC, we compared the disconnected case and the connected case, as described in the Materials and Methods section. In the disconnected case, we numerically simulated the responses of single spiking LPTCs isolated from other cells to the PD motion stimulus only presented in the ipsilateral visual hemi-field. In the connected case, we numerically simulated the responses of single spiking LPTCs to the in-phase and out-of-phase motion stimuli.

**Figure 2:**
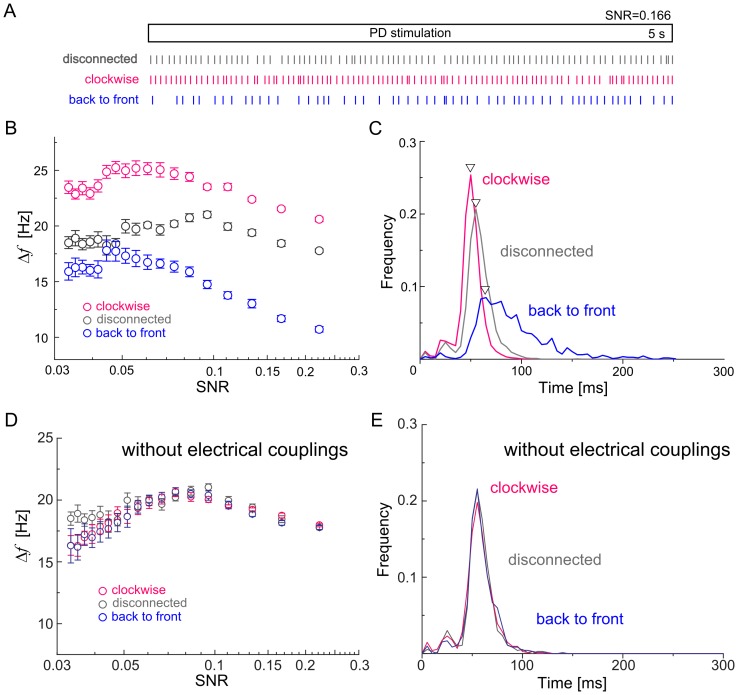
Activities of the H2 cell strongly depend on interhemispheric couplings between H2 and contralateral HSE. Gray in all figures: Responses of the H2L cell to the ipsilateral PD motion stimulus in the disconnected case. Red and blue: Responses of the H2L cell to the clockwise and back-to-front motion stimuli in the connected case (A, B and C) and the case without the interhemispheric electrical couplings (D and E). A: Raster plots showing locations of action potentials of the H2L cell in time for a single trial (SNR = 0.166). B: Differences in mean firing rate from spontaneous activity in the H2L cell in response to these motion stimuli with different noise levels. The abscissa indicates the signal-to-noise ratio of motion stimuli. The ordinate indicates difference between firing rates during stimulations and spontaneous activity. (mean
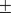
SEM, 8 trials) C: ISI distributions of the H2L cell in response to PD motion stimuli (SNR = 0.166). D: Differences in mean firing rates of the H2L cell without the interhemispheric electrical couplings. (mean
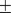
SEM, 8 trials) E: ISI distributions of the H2L cell without the electrical couplings. (SNR = 0.166). As shown in B and C, although the H2L cell directly faces the PD motion stimuli in the clockwise and back-to-front cases, the activity and regularity of the H2L cell for the clockwise motion stimulus are higher than those of the back-to-front stimulus because of the modification by contralateral LPTC activities. If the interhemispheric electrical couplings are only cut off and other connections remain in the bilateral network, as revealed in D and E, the activity and regularity of the H2L cell in this case is almost same as that in the disconnected case. Thus, these results suggest that the interhemispheric electrical couplings are a key factor to determining the responsive characteristics of the H2 cell to the binocular motion stimuli.

Figure 2A shows the spike raster plots displaying the spike times of the H2L cell in these cases, and Figure 2B show the difference of the mean firing rate from spontaneous activity in the H2L cell as a function of the signal-to-noise ratio (SNR) (defined in the Materials and Methods section). We found that the difference in mean firing rate induced by the clockwise motion in the connected case is lager than in the disconnected case. In contrast, the difference in mean firing rate induced by the back-to-front motion in the connected case is smaller than in the disconnected case. Figure 2C shows the distributions of inter-spike intervals (ISIs) of the H2L cell. The ISI distribution for the clockwise motion in the connected case is sharper than in the disconnected case, whereas the ISI distribution for the back-to-front motion in the connected case is broader than in the disconnected case. Therefore, though, in all these cases, the H2L cell faces the same PD motion stimulus in the ipsilateral visual hemi-field, the activity and regularity of the H2L cell are modified by the chenges in the contralateral LPTCs activities depending on motion stimuli in the contralateral visual hemi-field. Moreover, to investigate the contribution of interhemispheric electrical couplings between the H2 cell and contralateral HSE cell to the selectivity of the H2 cell, we removed the interhemispheric electrical couplings from the detailed model and simulated the response of the H2L cell similarly to the above. As shown in Figure 2D and Figure 2E, the differences in mean firing rate and the ISIs of the H2L cell are not altered by the type of binocular motion in this situation. Therefore, we consider that the interhemispheric electrical coupling is a key factor in determining the responsive characteristics of the H2 cell to binocular motion stimuli. Our numerical results are in accord with in-vivo experimental results indicating that the H2 cells are more activated by an in-phase motion stimulus than by an out-of-phase motion stimulus [13].

### The in-phase motion stimulus enhances synchronization between spiking LPTCs

In the previous subsection, we focused on modifications of single-cell activities depending on contralateral motion stimuli. Here, we tried to determine whether a combination of motion stimuli presented in the left and right visual hemi-fields affects the synchronization between spiking LPTCs. For this purpose, we calculated the cross-correlation between the firing rates of two spiking LPTCs (see the Materials and Methods section for details).

**Figure 3:**
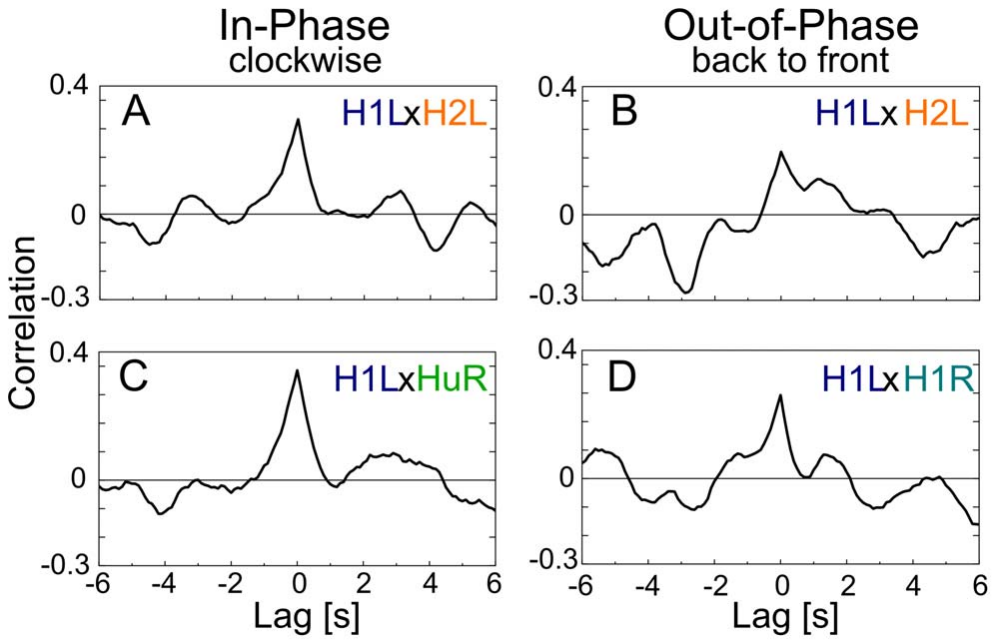
Change in firing-rate correlations between two spiking LPTCs depending on binocular stimuli. Left column: In-phase motion stimulus (clockwise). Right column: Out-of-phase motion stimulus (back-to-front). A and B: Cross-correlations between the H1 and H2 cells located in the left hemisphere. C: Cross-correlation between the H1 and Hu cells separately located in two hemispheres. D: Cross-correlation between two H1 cells separately located in two hemispheres. Note that the two LPTCs in each pair shown in these graphs receive PD motion stimuli in the in-phase and out-of-phase cases, respectively. The peak of the cross-correlations of the H1 and H2 cells at a lag of 0 sec for the in-phase motion stimulus is higher than that of the out-of-phase motion stimulus. Whereas the responsiveness of single H1 and Hu cells does not change with the contralateral motion stimuli (Supplements 2 and 3), the peak of the cross-correlations of these for the in-phase motion stimulus is higher than that of the out-of-phase motion stimulus.

Figures 3A and Figure 3B show the cross-correlation between the H1L and H2L cells located in the left hemisphere when presenting clockwise and back-to-front motion stimuli, respectively. In both these cases, each of the cells is exposed to the PD motion stimulus in the left visual hemi-field. When the two cells are exposed to the clockwise motion stimulus, the peak of the cross-correlation at a lag of 0 msec is higher than the peak that occurs with the back-to-front motion stimulus. This is because the out-of-phase motion stimulus decreases both the firing rate and regularity of the H2 cell (see Figure 2).

Figure 3C shows the cross-correlation between the H1L and HuR cells separately located in the two hemispheres when presenting the clockwise motion stimulus, and Figure 3D shows the cross-correlation between the H1L and H1R cells separately located in the two hemispheres when presenting the back-to-front motion stimulus. In both cases, each cell is exposed to the PD motion stimulus presented in the ipsilateral visual hemi-field. Thus, as shown in Figure S2 and Figure S3, these cells have identical differences in mean firing rate from the spontaneous activity and identical ISI distributions. However, as shown in Figure 3C and Figure 3D, the peak of the cross-correlation between the firing rates of the H1L and HuR cells at a lag of 0 msec when presenting the clockwise motion stimulus is slightly higher than that of the cross-correlation between the firing rates of the H1L and H1R cells when presenting the back-to-front motion stimulus. Therefore, the synchrony of these cells is enhanced by the in-phase motion stimulus. The numerical results suggest that the H1 and Hu cells, whose single-cell activities are independent of the motion stimuli presented in the contralateral visual hemi-field, could represent information on the binocular motion stimuli through their synchrony.

### Synchronous population activities of the bilateral LPTCs network represent binocular motion stimuli

In the previous subsections, we examined the modifications of the single-cell activities and synchronies in spiking LPTCs in relation to binocular motion stimuli. Here, we use principal component analysis (PCA) to reveal the properties of population activities of spiking LPTCs in response to four different motion stimuli. First, we calculated the mean firing rates of six spiking cells and construct a set of six-dimensional firing rate vectors (see the Materials and Methods section for details). Then, by applying PCA to the set of firing rate vectors, we projected them onto a two-dimensional space spanned by the first and second principal components, PC1 and PC2. PC1 and PC2 were found by calculating the two eigenvectors associated with the first- and second-largest eigenvalues of the correlation matrix obtained from the firing rate vectors. Thus, the principal components represent highly correlated or synchronous activity patterns of the spiking LPTCs. This analysis incorporated the correlation analysis carried out in the previous subsection.

**Figure 4:**
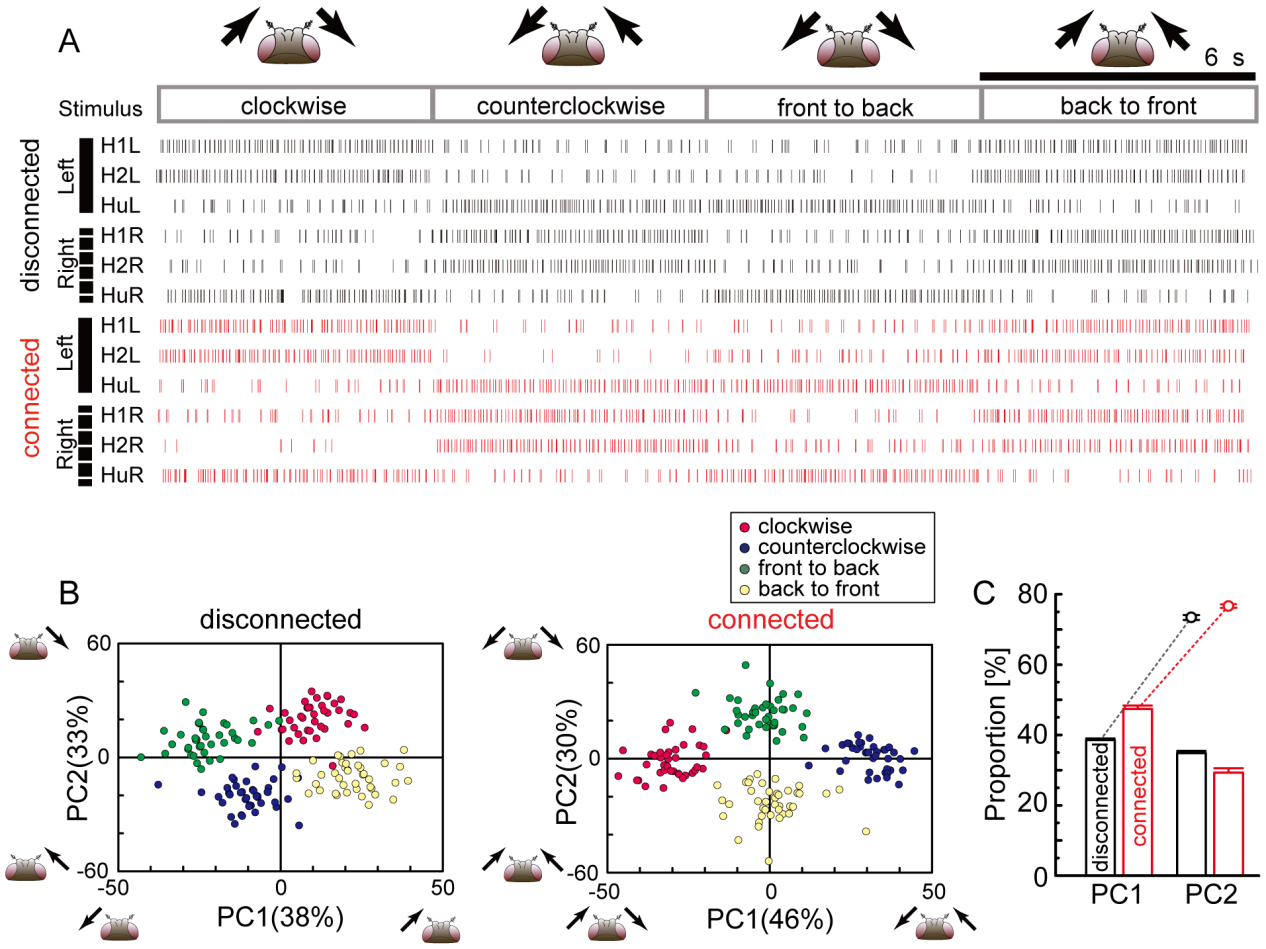
Coding properties of a population of LPTCs in a bilateral network. A: Raster plots showing locations of action potentials of all six spiking LPTCs in time for a single trial with the four different stimuli. Red plots indicate responses of spiking LPTCs in the connected case, and black plots present responses of spiking LPTCs in the disconnected case. B: Principal component analysis (PCA) for population activities shown in A. The firing rate vectors are projected onto a two-dimensional space spanned by the first and second principal components, PC1 and PC2. Colors indicate different stimuli. In the disconnected case, four clusters of the firing rate vectors corresponding to the four different stimuli are respectively separated into the four quadrants, whereas in the connected case, clusters of the firing rate vectors corresponding to the in-phase and out-of-phase stimuli are respectively distributed along the PC1 and PC2 axes. C: Contribution ratio of PC1 and PC2 (bars) and cumulative contribution ratio (dots) in the connected and disconnected cases. (mean
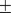
SEM, 10 trials).

The upper and lower parts of Figure 4A show examples of simulated spike sequences of six spiking LPTCs (denoted by raster plots) in the disconnected and connected cases for four different motion stimuli. Figure 4B shows the results obtained by applying PCA to these simulated spike sequences. In the disconnected case, four clusters of firing rate vectors corresponding to four different stimuli are respectively separated into the four quadrants in the PC1–PC2 space (left panel in Figure 4B). Clusters corresponding to the clockwise and back-to-front motion stimuli are distributed within the right half plane of the PC1–PC2 space. These two stimuli share the same visual motion in the left visual hemi-field. On the other hand, the clusters of the counterclockwise and front-to-back motion stimuli are distributed within the left half plane of the PC1–PC2 space. These two stimuli also share the same visual motion in the left visual hemi-field. Therefore, PC1 represents a population activity coding the left monocular visual motion. In the same way, each pair of clusters distributed within the upper or lower half plane of the PC1–PC2 space corresponds to the same visual motion in the right visual hemi-field. Thus, PC2 represents a population activity pattern that codes the right monocular motions. This result is trivial because in the disconnected case, the cells are isolated from each other without lateral connections. Supplement 3 shows the values of each element of PC1 and PC2 in five trials of numerical simulations with different random seeds for noise, and it also presents what each principle component codes in the five trials. It shows that PC1 and PC2 are randomly assigned to either left or right monocular motion. Thus, there is no eye dominance in the disconnected case. In the connected case, on the other hand, two pairs of clusters corresponding to the in-phase and out-of-phase motion stimuli are distributed along the PC1 and PC2 axes, respectively (right panel in Figure 4B). Therefore, the PC1 and PC2 represent population activity patterns that code the in-phase and out-of-phase motions, respectively. We also found that PC1 and PC2 stably represent the in-phase and out-of-phase motions with different random seeds for noise (Figure S3). Figure 4C shows the contribution ratio of PC1 and PC2 and the cumulative contribution ratio in the connected and disconnected cases. The difference between the contributions of PC1 and PC2 in the connected case is larger than those of the disconnected case.

To check whether or not the neuronal morphologies affect on the coding properties, we carried out an additional simulation under conditions in which the length of each LPTC is two-third that of the original model. We obtained the same results as those obtained by the original model (Figure S4). Therefore, we speculate that the neuronal morphologies do not strongly affect on the coding properties.

### Effect of interhemispheric electrical couplings between the H2 cell and the contralateral HSE cell on population coding in the bilateral LPTC network

Here, we evaluated the effect of the interhemispheric electrical coupling between the H2 cell and contralateral HSE cell on population coding in the bilateral network. We carried out numerical simulations on the detailed model using several different conductances of the interhemispheric electrical coupling, and we applied PCA to the simulated spike sequences, as in Figure 4. We used four different conductances: 0, 33.3, 50 and 100 nS. The parameters used in this simulation, except for the electrical coupling, were the same as in the previous simulations.

**Figure 5:**
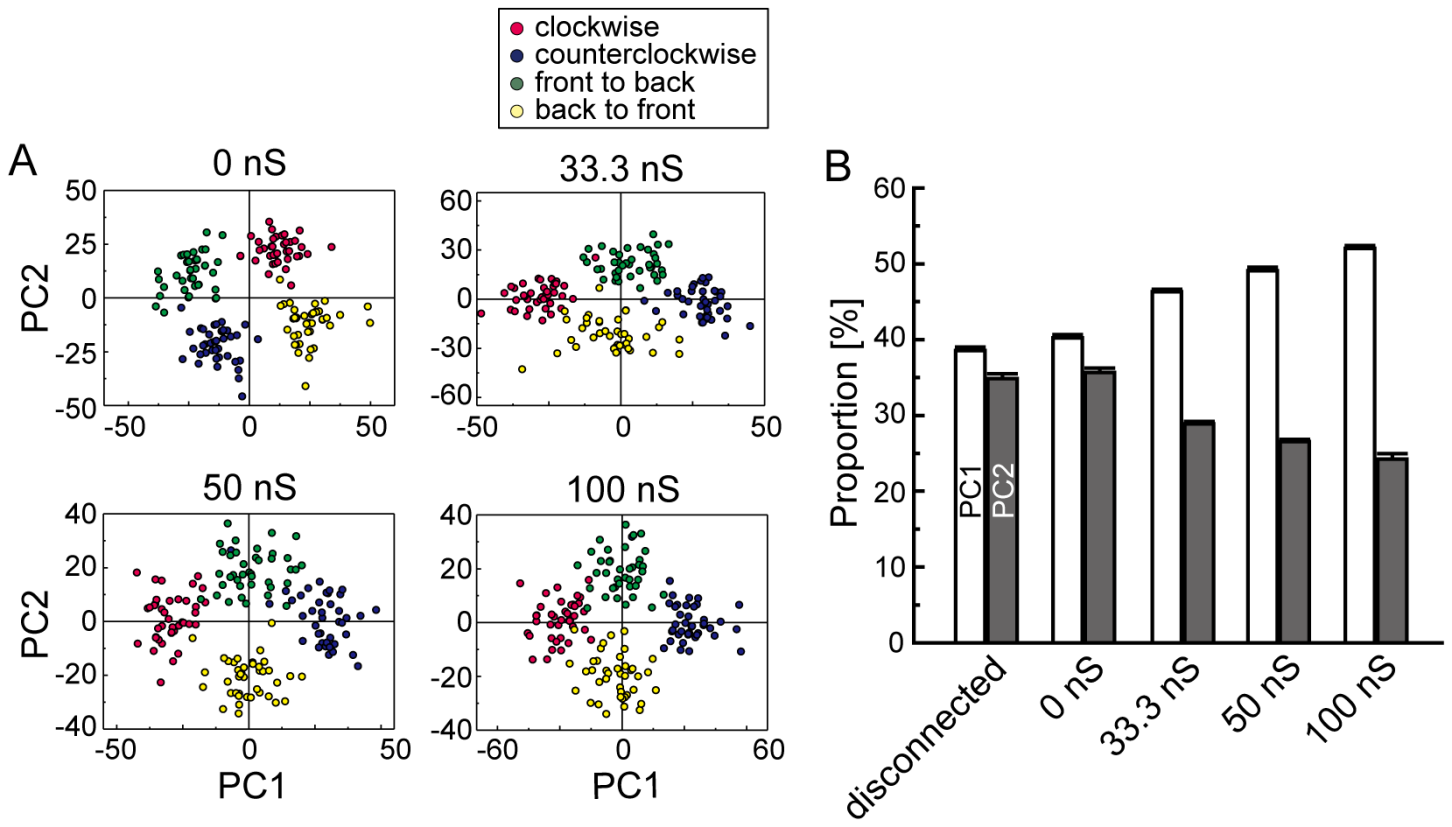
Interhemispheric couplings between H2 and HSE cells mainly affect the coding properties of population activities. A: PCAs for population activities using different values of the conductance of the electrical coupling. As in Fig. 4B, the firing rate vectors are projected onto a two-dimensional space spanned by PC1 and PC2. Colors indicate different stimuli. B: Contribution ratio of PC1 and PC2 as a function of the conductance of the electrical coupling. For comparison, the contribution ratio in the disconnected case is superimposed on this graph. As the conductance of the electrical coupling increases, the difference between contribution rates of PC1 and PC2 becomes larger. In the disconnected case and the case with the electrical coupling of 0 nS, the differences are relatively small, and four clusters of population activities corresponding to the four different stimuli are separated into the four quadrants, as shown in Figs. 5A and 4B. (mean
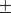
SEM, 10 trials).

As shown in Figure 5A, in the case of no electrical coupling (0 nS), four clusters of firing rate vectors corresponding to four different motion stimuli are respectively separated into the four quadrants of the PC1–PC2 space. This result conforms to that of the disconnected case shown in Figure 4B. Furthermore, as shown in Figure 5B, the difference in the contribution ratios of PC1 and PC2 is relatively small compared with those of the other nonzero cases, which is also similar to the disconnected case. When the electrical coupling between the H2 and HSE cells exists, two pairs of clusters corresponding to the in-phase and the out-of-phase motion stimuli are respectively distributed along the PC1 and PC2 axes (Figure 5A). The difference between the contribution ratios of PC1 and PC2 increases with the conductance of the electrical coupling (Figure 5B). These results suggest that the interhemispheric electrical coupling between the H2 and HSE cells strongly contributes to the coding properties of a population of LPTCs in the network.

### Properties of population coding in the detailed model are conserved in the reduced model

**Figure 6:**
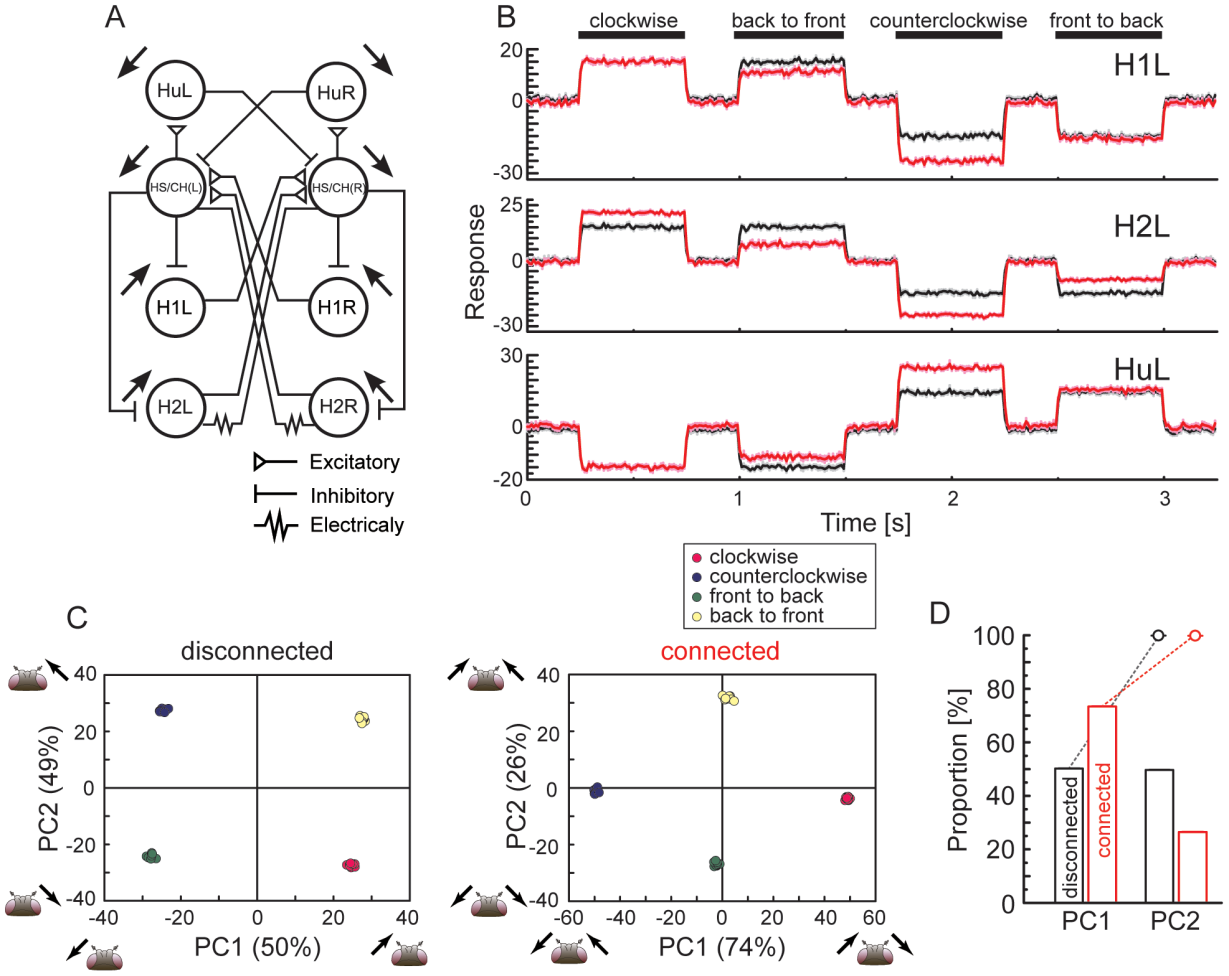
Coding properties are conserved in the reduced model. A: Summary diagram of the reduced model. For simplicity, in each hemisphere, five graded-potential cells are merged into a single cell named HS/CH. All cells are described using the McCulloch-Pitts model instead of the conductance-based model. B:Activities of three LPTCs corresponding to the spiking cells on the left side in response to the four different stimuli. Black and red lines denote the disconnected and connected cases, respectively. C: PCA for population activities shown in B. By applying PCA to activity vectors whose elements correspond to activities of six LPTCs on the left and right hemispheres, we projected the activity vectors onto a two-dimensional space spanned by the first and second principal components, PC1 and PC2. Colors indicate different stimuli. In the disconnected case, four clusters of the activity vectors corresponding to the four different stimuli are respectively separated into the four quadrants, whereas in the connected case, clusters of the activity vectors corresponding to the in-phase and out-of-phase stimuli are respectively distributed along the PC1 and PC2 axes. D: Contribution ratio of PC1 and PC2 (bars) and cumulative contribution ratio (dots) in the connected and disconnected cases. (mean
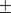
SEM, 10 trials).

To check whether or not the numerical results are specific to the network model we constructed, we construct a reduced model and tried to reproduce the results of Figure 4. To simplify the structure of the LPTC network, in each hemisphere, we merged five graded-potential neurons, which are coupled through electrical synapses, into a single neuron named HS/CH, as shown in Figure 6A. Furthermore, to simplify the activity properties of cells, we described all LPTCs in the network by using the McClloch-Pitts model instead of the conductance-based model (see the Materials and Methods section for details).

The left and right panels of Figure 6B show examples of the activities of three LPTCs corresponding to the spiking cells in the left hemisphere in response to the four different motion stimuli in the connected and disconnected cases. Figure 6C shows results obtained by applying PCA to a set of six-dimensional state vectors consisting of the responses of six spiking LPTCs in both hemispheres. In the disconnected case (left panel in Figure 6C), four clusters of state vectors corresponding to four different motion stimuli are respectively separated into the four quadrants of the PC1–PC2 space, whereas in the connected case (right panel in Figure 6C), two pairs of clusters corresponding to the in-phase and the out-of-phase motion stimuli are distributed along the PC1 and PC2 axes. Figure 6D shows the contribution ratio of PC1 and PC2 and the cumulative contribution ratio in the connected and disconnected cases. The difference between the contributions of PC1 and PC2 in the connected case is larger than in the disconnected case. Figure S5 shows the values of each element of PC1 and PC2. The principal components of the reduced model qualitatively correspond to those of the detailed model. Therefore, the results obtained from the reduced model qualitatively conform to those of the detailed model in Figure 4.

## Discussion

### Summary of Results and Conclusion

We investigated the cooperative behavior of the LPTCs underlying the integration of binocular motion information and the information representation in the bilateral LPTC network through numerical simulations. First, we showed that the cooperative activities of cells in the bilateral network via interhemispheric couplings, especially interhemispheric electrical couplings, could account for the in-phase sensitive response of the H2 cells that was previously reported (Figure 2). Moreover, the results of cross-correlation analyses suggested that the other spiking LPTCs, H1 and Hu, might be involved in representing binocular motion in a manner that is a synchrony of these activities (Figure 3). We also applied PCA to the firing rates of all spiking LPTCs and found that when the LPTCs are isolated from each other; two orthogonal patterns of correlated population activities given by PC1 and PC2 represent the monocular motions, whereas when the LPTCs are connected to each other, two orthogonal pattens of correlated population activities respectively represent the in-phase and out-of-phase motions, and the population activity is more sensitive to the in-phase motion stimuli (Figure 4). Moreover, we found that the interhemispheric electrical couplings strongly influence these population-coding properties (Figure 5). Finally, we confirmed the generality and robustness of these results by using a reduced model (Figure 6).

### Intuitive explanation of the binocular motion integration in the bilateral network

**Figure 7:**
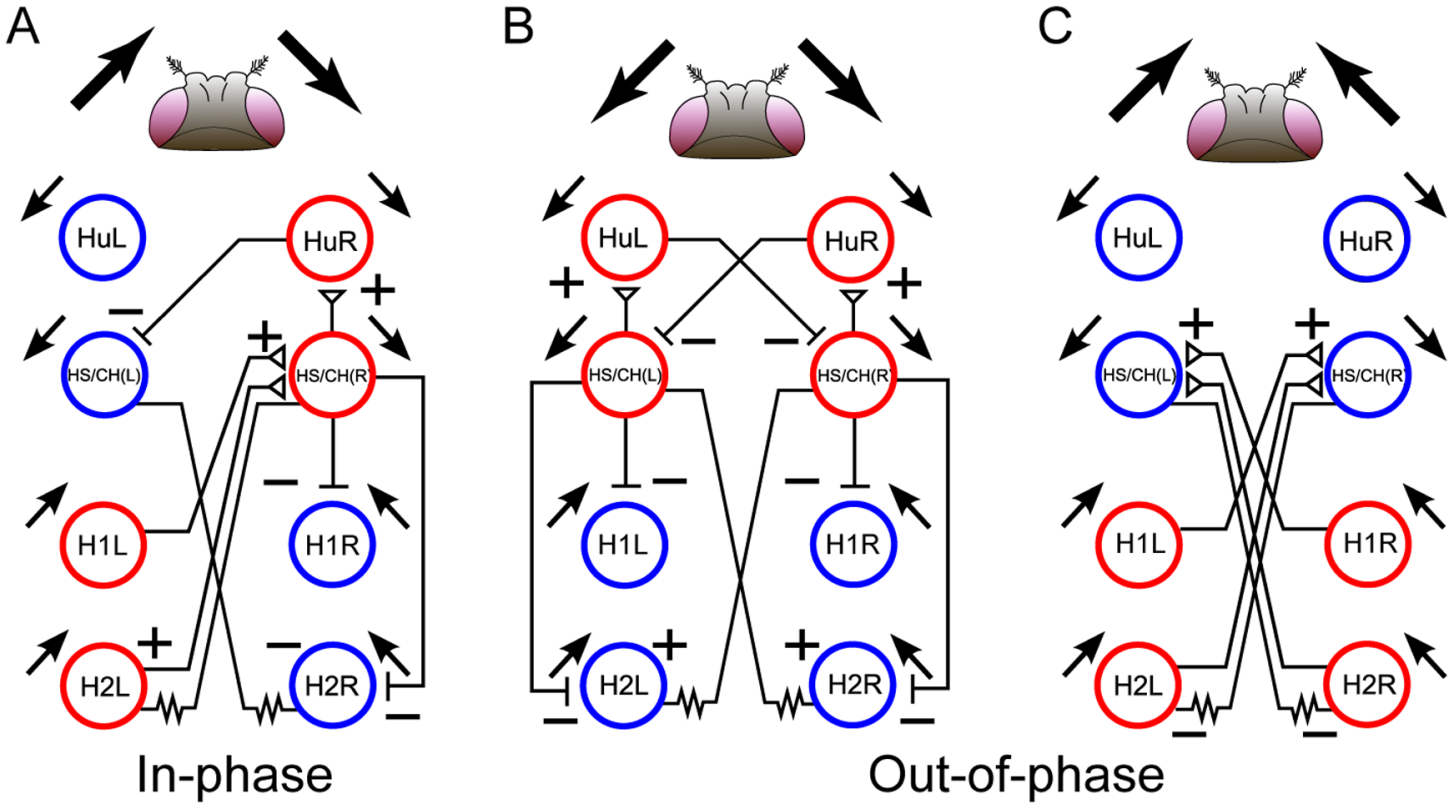
Mechanisms of binocular integration of visual information. Red and blue circles indicate depolarized and hyperpolarized cells in response to ipsilateral PD and ND motion stimuli, respectively. A: In the in-phase case, the cells responding to ipsilateral PD stimuli receive excitatory inputs from contralateral and ipsilateral LPTCs, and the cells responding to ipsilateral ND stimuli receive inhibitory inputs from the other LPTCs. The cells integrate the in-phase motion stimuli through their cooperative behavior. B, C: In the out-of-phase case, some cells responding to ipsilateral PD stimuli receive inhibitory inputs from the other LPTCs, and some cells responding to ipsilateral ND stimuli receive excitatory inputs from the other LPTCs. Thus, there is a frustration in the out-of-phase case because the activities of the neurons interfere with the mutual interactions.

Let us try to intuitively understand the cooperative behavior of the LPTCs underlying binocular motion integration by referring to the reduced model. Figure 7 illustrates the activity patterns of the cells responding to the in-phase and out-of-phase motion stimuli and the synaptic connections of the network. In the case of the in-phase motion stimulus, the polarity of each cell depending on the PD motion matches the polarity of each lateral synaptic connection (Figure 7A), resulting in an enhancement of each cell's sensitivity to the PD motion. In this situation, cells cooperatively integrate the motion information in the bilateral network. On the other hand, in case of the out-of-phase motion stimulus, the polarity of each cell depending on its PD motion is somewhat mismatched to the polarity of the lateral synaptic connections (Figure 7B and Figure 7C), resulting in a decrease in sensitivity. This mechanism can account for the increase in the response of some LPTCs during the in-phase motion stimulation reported in previous in vivo experiments [8]–[10], [13], [29]. Furthermore, it is known that mismatches between cell polarity and lateral synaptic polarity, which are referred to as frustration, induce asynchronous cell activities in a general class of networks. Thus, this mechanism can also account for the greater synchrony among LPTCs in response to in-phase motion compared with the response to the out-of-phase motion (Figure 3 and Figure 4).

### Reliability of modeling with the ML model

Through in vivo experiments, Farrow et al. [13] suggested the possibility that the interhemispheric electrical coupling between H2 and contralateral HSE cells is involved in the flow field selectivity of H2 cells. Moreover, they indicated through numerical simulations that the electrical couplings can quantitatively account for the selectivity of the H2 cell. In the simulations, they used a simplified neural network of the horizontal LPTCs, which consisted of four horizontal cells, H1, H2, HS and CH cells in each hemisphere. In contrast, our neural network model (the detailed model) has a more anatomically accurate structure, in which we modeled all of horizontal LPTCs that include cells ignored in Farrow et al. [13]. We demonstrated that the in-phase sensitive response of H2 cells can also be qualitatively reproduced by our detailed model and that it can be accounted for by the interhemispheric electrical coupling (Figure 2). Our numerical results are consistent with the results of Farrow et al. [13], and this consistency ensures the reliability of the results obtained from our model.

Farrow et al. [13] used the Hodgkin-Huxley model for the spiking LPTCs. At present, however, the properties and distribution of the ion-channels of spiking LPTCs are not well understood. Hence, the Hodgkin-Huxley model is not the only way to model spiking LPTCs. In this paper, we used the Morris-Lecar (ML) model for the spiking LPTCs in the detailed model. The ML model, which has a two-dimensional state space, shares a common bifurcation structure with other high-dimensional Hodgkin-Huxley type models classified into types I and II, and it can reproduce electrical responsiveness of typical neurons. Therefore (invoking Occam's razor), in circumstances where the properties of the membrane ion-conductances of the cell that we want to model are unknown, a lower dimensional model with high explanatory power should be used. However, in the ML model, the firing rate is limited to less than 30 Hz. The firing rate of the real spiking LPTCs, for example, the H2 cell, is more than 50 Hz, or even 100 Hz in some circumstances. Thus, the ML model cannot adequately reproduce the firing rate of spiking LPTCs. Despite this, the numerical results obtained from the ML model qualitatively conform to results observed in vivo [10], [13]. Moreover, as shown in Figure 6, we confirmed that the results of simulation are not dependent on the conductance-based model we used by testing the more simplified model consisting of McCulloch Pitts units. According to the consistent results obtained from the two models, we conclude that the spiking mechanism based on the properties of the membrane ion-channels do not affect properties of population coding in the network sensitively. Accordingly, we consider that the ML model is able to capture the mechanism of binocular motion integration.

### Potential of synchronized coding of binocular motion in spiking LPTCs other than H2 cells

In the detailed model, we demonstrated that the firing rate and regularity of single H1 and Hu cells, unlike H2 cells, are not influenced by motion stimuli in the contralateral visual hemi-field (see Figure S1 and Figure S2). However, we showed that the correlations between these cell activities are enhanced by the in-phase motion stimulus (Figure 3). From a structural viewpoint, there is a major difference between H2 and the other horizontal spiking LPTCs in the manner of their receiving inputs from the contralateral LPTCs. H2 cells directly receive input via the interhemispheric electrical coupling from the contralateral cell;therefore, the activity of H2 cells is strongly influenced by the contralateral motion stimulus. H1 and Hu cells, on the other hand, indirectly receive input from the contralateral hemisphere via the ipsilateral graded-potential cells, and the input is not strong enough to change the firing rate or regularity. The spike timings of H1 and Hu cells are entrained with each other through the indirect and weak interaction, and this makes the correlation of the activities of these cells dependent on the binocular motion. It is theoretically conjectured that the weak interaction, which is too weak to change phase trajectories in oscillations, enhances the synchronization of spikes across neurons [37], [38]. This result suggests that H1 and Hu cells, unlike H2 cell, represent the binocular motions by using not the individual cell activity but the correlation of activities. Little is known about the responsivity of H1 and Hu cells to binocular motion. To reveal the representation of binocular motion in these cells, the responsivity of these LPTCs to binocular motion will have to be recorded electro-physiologically.

### Significance of sensitive response to in-phase motion in optomotor response

What are the functional roles of the sensitive response to the in-phase motion? It has been reported that neurons sensitive to in-phase motion exist in other species, for instance, descending neurons (DNVII_1_) in the honeybee [39]. It is also known that many species of insect, crustacean, and mammal have the ability to stabilize retinal images by moving their eyes, head or whole body to compensate for their movements through the environment [20], [40], [41]. This motor action is referred to as the optomotor response. It can be thought that the neurons sensitive to the in-phase motion provide the most important cue for the optomotor response, because retinal image motions evoked by perturbed movements of the observer's head and body are the in-phase motion in most cases.

### Importance of our analysis method

A large number of studies have sought to reveal the coding properties of neural populations by using simultaneous multi-neuronal recordings and statistical techniques [42]–[45]. However, there is a limit on the number of neurons that can be simultaneously recorded in vivo. Thus, we must infer the population-coding properties of a whole local network from partial data. The limitations of such a measurement make it hard to understand the population coding in the whole local network.

On the other hand, researchers are using advanced genetic tools to get a complete picture of synaptic interactions in the whole brain of the fly [46]. If the synaptic interactions in local networks can be completely identified, we can construct accurate models of these local networks. Then, by combining numerical simulations of the network model with statistical techniques, we can elucidate the population-coding properties of the whole local network. Moreover, by altering the conductance of a particular synapse in the network model, we can evaluate the contribution of the synapse to the information processing or representation of the network. In this paper, we showed that interhemispheric electrical couplings play a key role in the integration of binocular motion information. Although compared with chemical synapses, much less is known about how electric couplings contribute to information processing in a neural network, it has recently become recognized that the electric couplings play a significant role in information processing in the local network [47]. Our study provides an important clue to understanding the functional role of electrical couplings in visual information processing. Moreover, unlike multi-neuronal recording approaches, we can use our approach to answer a big question about how the computation is implemented with neural interactions

## Supporting Information

Figure S1Activities of the H1L cell in response to PD motion stimuli are not modified by contralateral LPTC activities. (gray) Responses of the H1L cell to the ipsilateral PD motion stimulus in the disconnected case. (red) Responses of the H1L cell to the clockwise motion stimulus in the connected case. (blue) Responses of the H1L cell to the back-to-front motion stimulus in the connected case. A: Differences in mean firing rate from spontaneous activity in the H1L cell in response to these motion stimuli with different noise levels. The abscissa is the signal-to-noise ratio of the motion stimuli. The ordinate is the difference between firing rates during stimulation and spontaneous activity. (mean
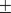
SEM, 8 trials) B: ISI distributions of the H1L cell in response to PD motion stimuli (SNR = 0.166). The activity and regularity of the H1L cell when the facing of the clockwise motion stimulus is almost the same as that of the back-to-front stimulus.(TIF)

Figure S2Activities of the HuL cell in response to PD motion stimuli are not modified by contralateral LPTC activities. (gray) Responses of the HuL cell to the ipsilateral PD motion stimulus in the disconnected case. (red) Responses of the HuL cell to the counterclockwise motion stimulus in the connected case. (blue) Responses of the HuL cell to the front-to-back motion stimulus in the connected case. A: Differences in mean firing rate from spontaneous activity in the HuL cell in response to stimuli with different noise levels. The abscissa indicates the signal-to-noise ratio of motion stimuli. The ordinate indicates differences between firing rates during stimulations and spontaneous ones. (mean
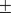
SEM, 8 trials) B: ISI distributions of the HuL cell in response to PD motion stimuli (SNR = 0.166). The activity and regularity of the HuL cell when the facing of the counterclockwise motion stimulus is almost same as that of the front-to-back stimulus.(TIF)

Figure S3 Each element of the first two principal components, PC1 and PC2, in five trials of numerical simulations for the detailed model with different random seeds for noise. The upper table is the disconnected case, and the lower table is the connected case. What each principle component codes in the five trials is presented on the margins of these tables. In the connected case, PC1 and PC2 stably represent the in-phase and out-phase motions, whereas in the disconnected case, PC1 and PC2 are randomly assigned to either left or right monocular motion.
(TIF)

Figure S4The neuronal morphologies do not affect on the population coding properties. A: Principal component analysis (PCA) for population activities. We analyzed population coding properties under conditions in which the length of each LPTC is two-third that of the original model. The firing rate vectors are projected onto a two-dimensional space spanned by the first and second principal components, PC1 and PC2. Colors indicate different stimuli. Clusters of the firing rate vectors corresponding to the in-phase and out-of-phase stimuli are respectively distributed along the PC1 and PC2 axes. This result is the qualitatively same as those shown in Figure 4B. B: Contribution ratio of PC1 and PC2 (bars) and cumulative contribution ratio (dots). (mean
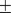
SEM, 10 trials).(TIF)

Figure S5 Each element of the first two principal components, PC1 and PC2, in five trials of numerical simulations for the reduced model with different random seeds for noise. The upper table is the disconnected case, and the lower table is the connected case. What each principle component codes in the five trials is presented on the margins of these tables. In the connected case, PC1 and PC2 stably represent the in-phase and out-phase motions, whereas in the disconnected case, PC1 and PC2 are randomly assigned to either left or right monocular motion.
(TIF)
