# Supplementary figures and images for: Cooperative Integration and Representation Underlying Bilateral Network of Fly Motion-Sensitive Neurons
Source: PLoS One. 2014 Jan 23;9(1):e85790. doi: 10.1371/journal.pone.0085790 (PMC3900430; doi:10.1371/journal.pone.0085790)

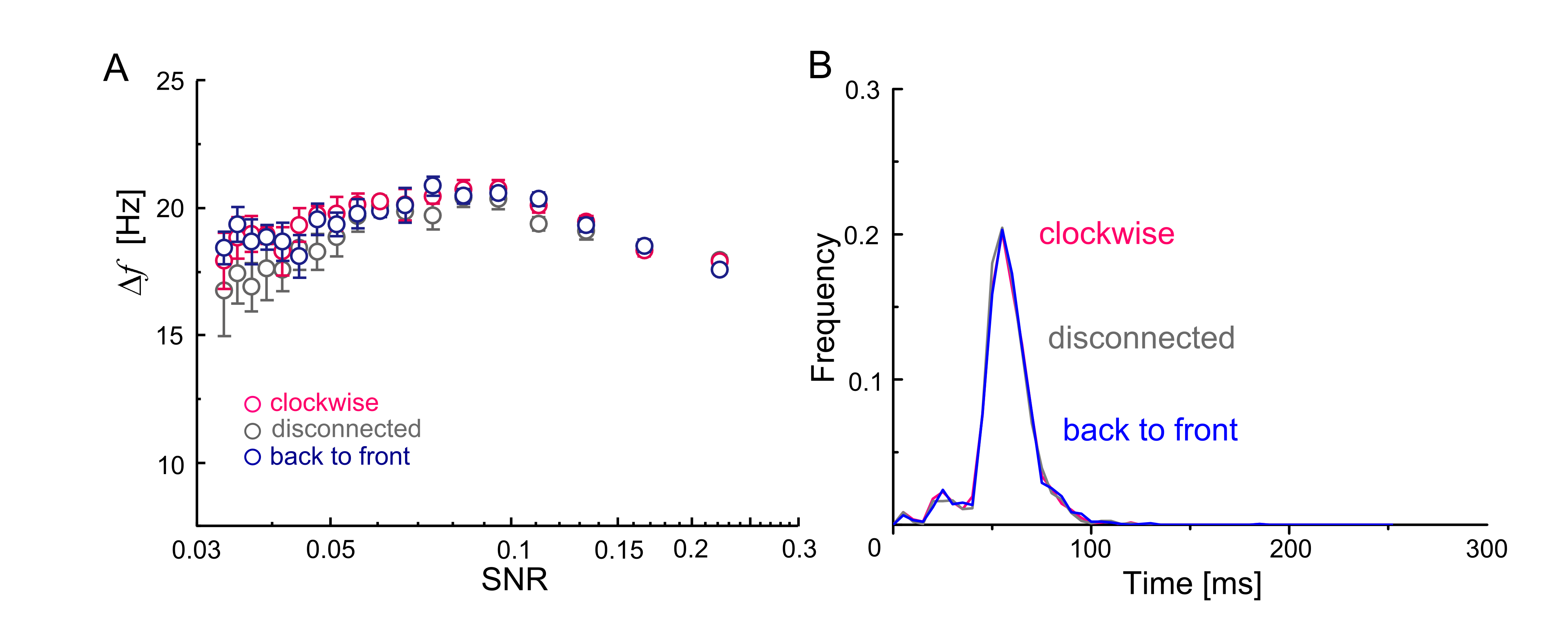

Supplement: Figure S1 — Activities of the H1L cell in response to PD motion stimuli are not modified by contralateral LPTC activities. (gray) Responses of the H1L cell to the ipsilateral PD motion stimulus in the disconnected case. (red) Responses of the H1L cell to the clockwise motion stimulus in the connected case. (blue) Responses of the H1L cell to the back-to-front motion stimulus in the connected case. A: Differences in mean firing rate from spontaneous activity in the H1L cell in response to these motion stimuli with different noise levels. The abscissa is the signal-to-noise ratio of the motion stimuli. The ordinate is the difference between firing rates during stimulation and spontaneous activity. (meanSEM, 8 trials) B: ISI distributions of the H1L cell in response to PD motion stimuli (SNR = 0.166). The activity and regularity of the H1L cell when the facing of the clockwise motion stimulus is almost the same as that of the back-to-front stimulus. (TIF) [file pone.0085790.s001.tif]

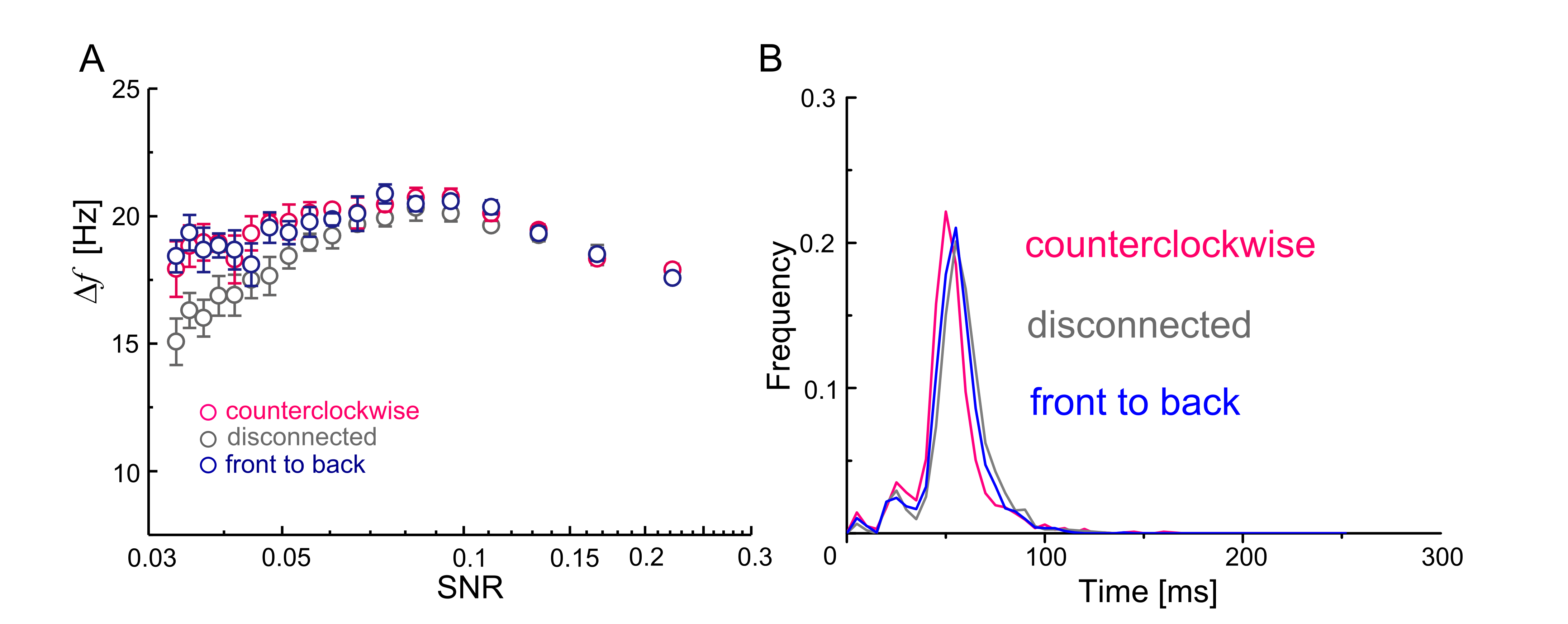

Supplement: Figure S2 — Activities of the HuL cell in response to PD motion stimuli are not modified by contralateral LPTC activities. (gray) Responses of the HuL cell to the ipsilateral PD motion stimulus in the disconnected case. (red) Responses of the HuL cell to the counterclockwise motion stimulus in the connected case. (blue) Responses of the HuL cell to the front-to-back motion stimulus in the connected case. A: Differences in mean firing rate from spontaneous activity in the HuL cell in response to stimuli with different noise levels. The abscissa indicates the signal-to-noise ratio of motion stimuli. The ordinate indicates differences between firing rates during stimulations and spontaneous ones. (meanSEM, 8 trials) B: ISI distributions of the HuL cell in response to PD motion stimuli (SNR = 0.166). The activity and regularity of the HuL cell when the facing of the counterclockwise motion stimulus is almost same as that of the front-to-back stimulus. (TIF) [file pone.0085790.s002.tif]

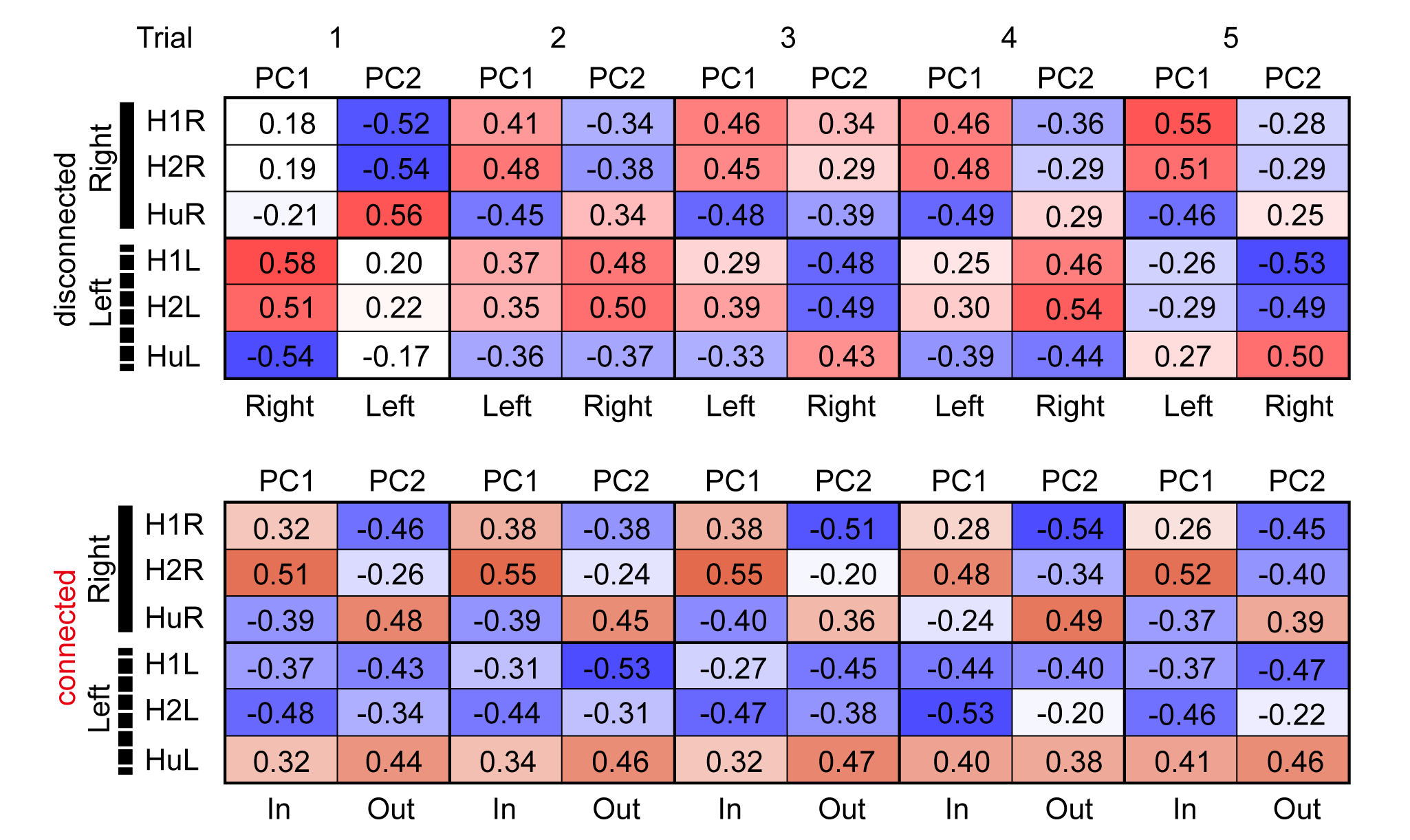

Supplement: Figure S3 — Each element of the first two principal components, PC1 and PC2, in five trials of numerical simulations for the detailed model with different random seeds for noise. The upper table is the disconnected case, and the lower table is the connected case. What each principle component codes in the five trials is presented on the margins of these tables. In the connected case, PC1 and PC2 stably represent the in-phase and out-phase motions, whereas in the disconnected case, PC1 and PC2 are randomly assigned to either left or right monocular motion. (TIF) [file pone.0085790.s003.tif]

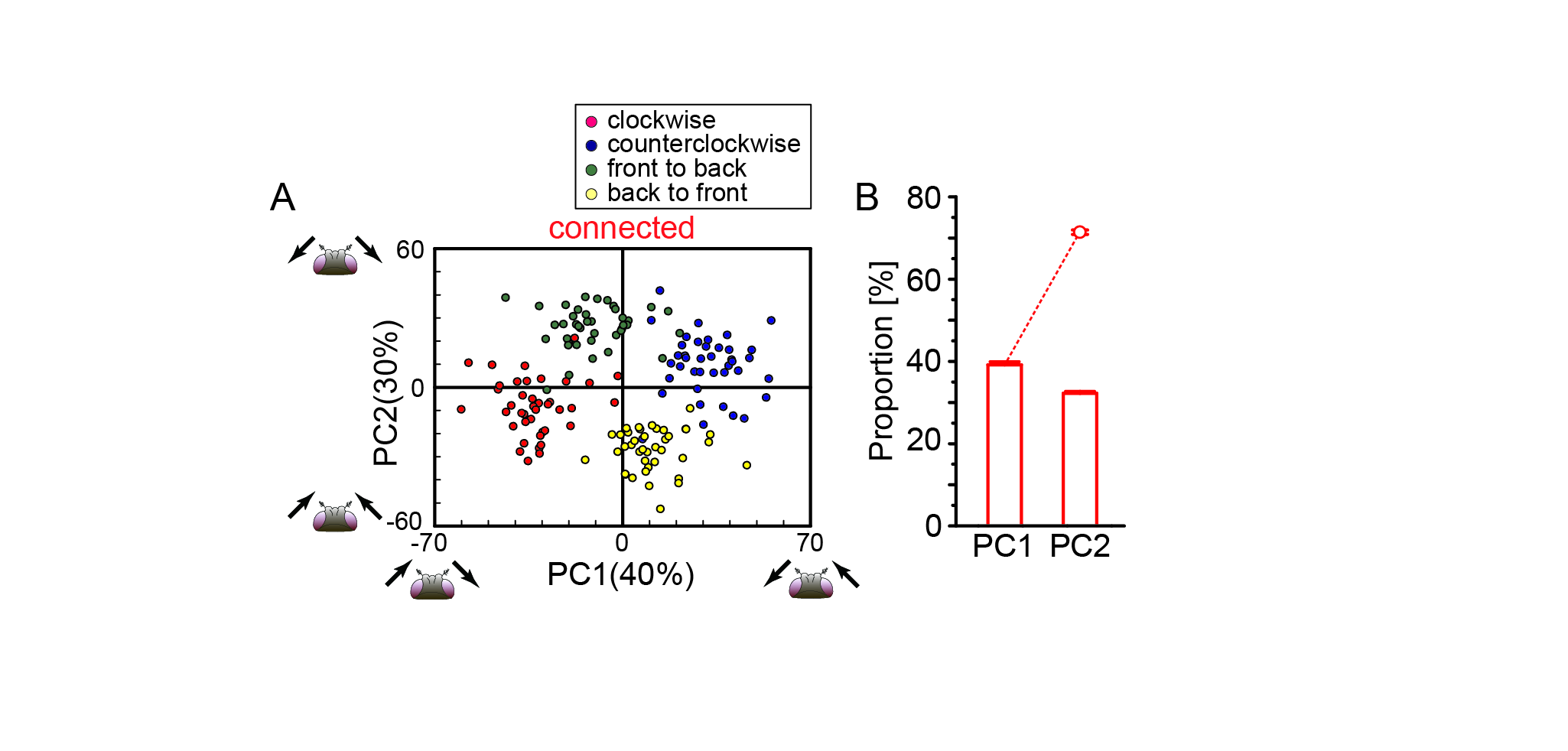

Supplement: Figure S4 — The neuronal morphologies do not affect on the population coding properties. A: Principal component analysis (PCA) for population activities. We analyzed population coding properties under conditions in which the length of each LPTC is two-third that of the original model. The firing rate vectors are projected onto a two-dimensional space spanned by the first and second principal components, PC1 and PC2. Colors indicate different stimuli. Clusters of the firing rate vectors corresponding to the in-phase and out-of-phase stimuli are respectively distributed along the PC1 and PC2 axes. This result is the qualitatively same as those shown in Figure 4B. B: Contribution ratio of PC1 and PC2 (bars) and cumulative contribution ratio (dots). (meanSEM, 10 trials). (TIF) [file pone.0085790.s004.tif]

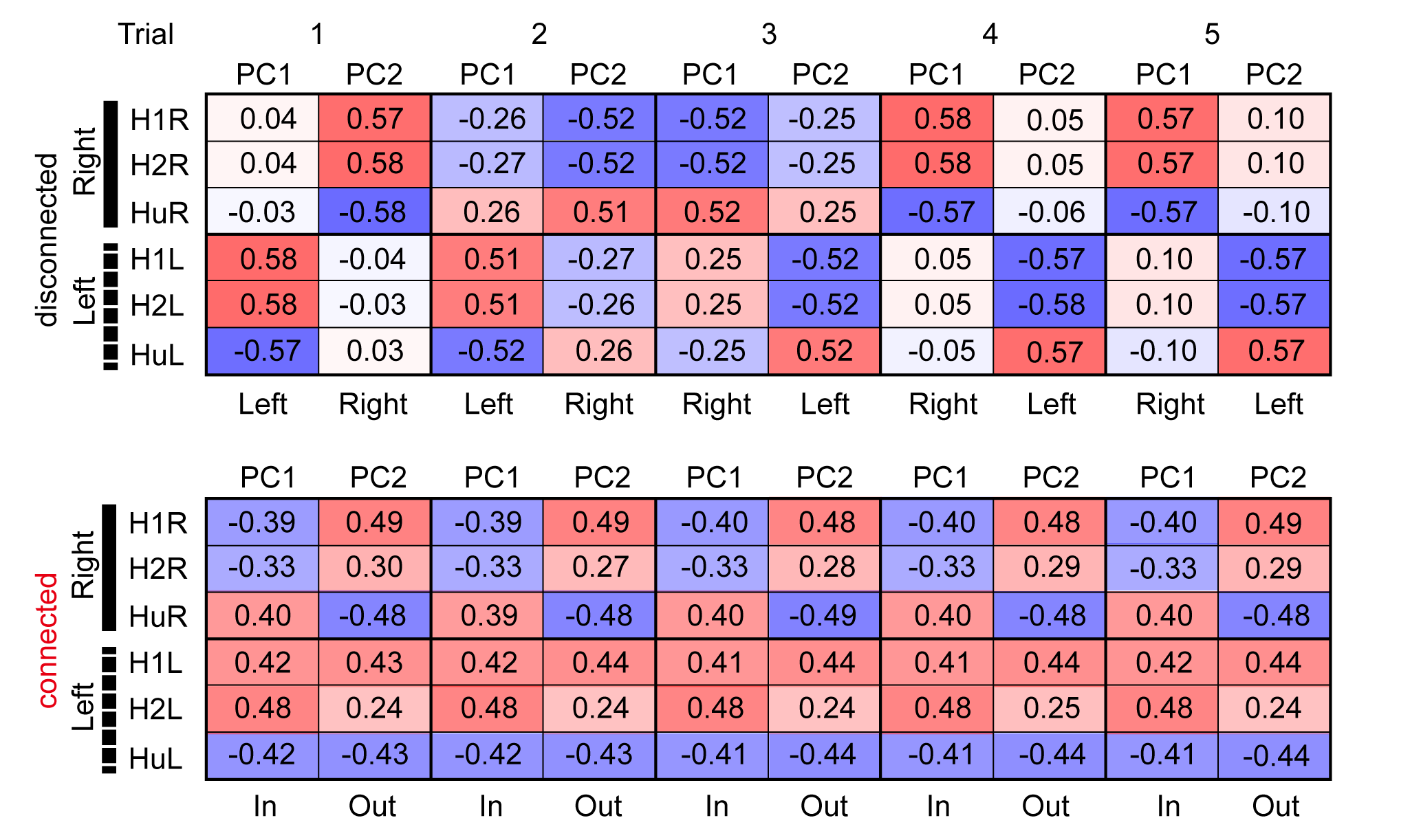

Supplement: Figure S5 — Each element of the first two principal components, PC1 and PC2, in five trials of numerical simulations for the reduced model with different random seeds for noise. The upper table is the disconnected case, and the lower table is the connected case. What each principle component codes in the five trials is presented on the margins of these tables. In the connected case, PC1 and PC2 stably represent the in-phase and out-phase motions, whereas in the disconnected case, PC1 and PC2 are randomly assigned to either left or right monocular motion. (TIF) [file pone.0085790.s005.tif]
